# Retrospective Analysis of the Equine Influenza Virus A/Equine/Kirgizia/26/1974 (H7N7) Isolated in Central Asia

**DOI:** 10.3390/pathogens5030055

**Published:** 2016-08-10

**Authors:** Kobey Karamendin, Aidyn Kydyrmanov, Marat Sayatov, Vitaliy Strochkov, Nurlan Sandybayev, Kulaysan Sultankulova

**Affiliations:** 1 Institute of Microbiology and Virology, 103 Bogenbay Batyr Str., Almaty 050010, Kazakhstan; kydyrmanov@yandex.kz (A.K.); ecovir@nursat.kz (M.S.); 2 Research Institute for Biological Safety Problems, Gvardeyskiy 080409, Zhambyl Region, Kazakhstan; vstrochkoV@biosafety.kz (V.S.); nurlan.s@mail.ru (N.S.); sultankul@biosafety.kz (K.S.)

**Keywords:** equine influenza, Central Asia, H7N7, sequence, phylogeny

## Abstract

A retrospective phylogenetic characterization of the hemagglutinin, neuraminidase and nucleoprotein genes of equine influenza virus A/equine/Kirgizia/26/1974 (H7N7) which caused an outbreak in Kirgizia (a former Soviet Union republic, now Kyrgyzstan) in 1977 was conducted. It was defined that it was closely related to the strain London/1973 isolated in Europe and it shared a maximum nucleotide sequence identity at 99% with it. This Central Asian equine influenza virus isolate did not have any specific genetic signatures and can be considered as an epizootic strain of 1974 that spread in Europe. The absence of antibodies to this subtype EI virus (EIV) in recent research confirms its disappearance as of the 1990s when the antibodies were last found in unvaccinated horses.

## 1. Introduction

Equine influenza (EI) is a widespread disease of horses caused by the influenza A virus. The virus causes upper respiratory infections of varying degrees of severity. The viruses currently circulating in horses worldwide belong to the H3N8 subtype consisting of two lineages—American and Eurasian [1]. EI virus (EIV) infections due to the H7N7 subtype have not been reported since the end of the 1970s [2]. There was only some serological evidence in the 1990s indicating the circulation of this EIV serotype in horses in Central Asia, and the H7N7 subtype is now considered to be no longer circulating in horses [3].

Historically, EIV caused an outbreak in Central Asia in 1992, but the virus was not genetically characterized then [4]. The 1992 EI outbreak might be related to the 1989 or 1990 EIV outbreaks in China [5] caused by the H3N8 subtype. A second documented outbreak occurred in 2007, concurrent with several outbreaks in China, Mongolia and India [6,7,8]. A third restricted outbreak occurred there in 2012. It was defined that two equine influenza H3N8 viruses, belonging to the H3N8 subtype Florida clade 2, caused the 2007 and 2012 EI outbreaks in Kazakhstan, which shares a common border with Kyrgyzstan [9].

Horse breeding is one of the most important agricultural branches in Central Asia. The local population has bred horses for thousands of years, and this region is considered to be the place where horses were first domesticated [10]. The large population of horses in Central Asia (more than 20 million) [11] provides a strong basis for sustained transmission and evolution of EIV in equine hosts.

Despite the serological evidence of circulation of the H7N7 equine influenza subtype in Central Asia, no EIV of this subtype was described in the scientific literature before this research. Sequence data of the only isolate was published in Genbank in 2012 and this was the first published isolate from all over Asia.

Here, we report the retrospective phylogenetic characterization of the hemagglutinin, neuraminidase and nucleoprotein genes of the equine influenza virus belonging to the H7N7 EIV serotype, which caused the outbreak in Kirgizia (a former Soviet Union republic, now Kyrgyzstan) in 1977.

The purpose of this study was to genetically characterize that isolate in comparison to all available H7N7 equine influenza viruses in public databases and figure out the potential source of the introduction of the virus to the Central Asian region in that period.

## 2. Results and Discussion

The phylogenetic analysis supported previously published data indicating the existence of two major H7N7 EIV sublineages: the prototype-like, and a group comprising other equine viruses ascending to the early American isolates of the mid-1960s [12]. In the latter group, divergence into two geographically based clusters, American and Eurasian, can be observed.

**Figure 1:**
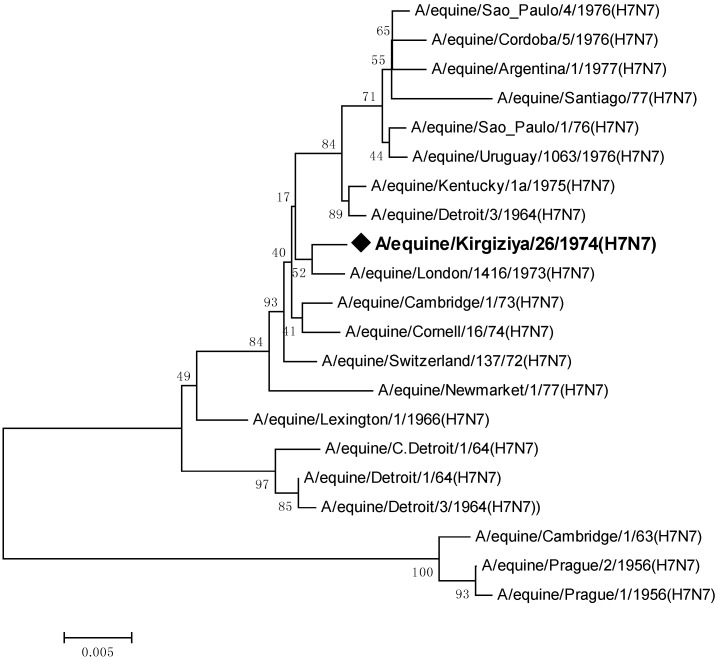
Phylogenetic analysis of isolate A/equine/Kirgizia/26/1974 (H7N7). An unrooted neighbor-joining tree of nucleotide sequences of the HA gene was generated, followed by 1000 bootstrap re-samplings.

Comparing the entire sequence of hemagglutinin (HA) gene of isolate A/equine/Kirgizia/26/1974 (H7N7) with all sequences from the 1970s available in the Genbank (Figure 1), we found that it belongs to the same branch of London/1973 isolated in Europe and is less similar to the American lineage.

Evolutionary distance analysis using MEGA 6.0 software has shown a high degree of identity, greater than 98% among viruses isolated after 1970s. As indicated earlier [13], the hemagglutinin of H7 EIV shows only limited variation, but the HA genes of the earliest strains of the virus are quite distinct from strains isolated later.

The strain Kirgizia/74 shared a maximum nucleotide sequence identity at 99% with virus London/1973. There were an eight-nucleotide difference between them and four of them were synonymous substitutions. Also, the Central Asian strain had a unique non-synonymous substitution N164D at antigenic site B (Table 1).

The virus Kirgizia/74 possessed a tetrabasic amino acid cleavage site RKKR/GLF separating the HA1 and HA2 domains, as in other EIVs of this subtype.

**Figure 2:**
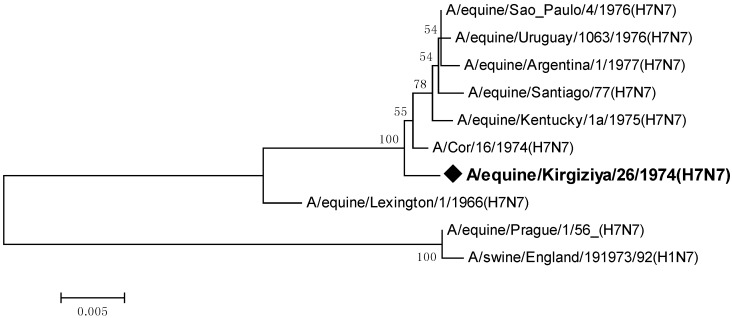
Phylogenetic tree of all available nucleotide sequences of neuraminidase gene of the H7N7 equine influenza viruses.

Also, we compared the neuraminidase gene sequence of the isolate under study with sequences available in public databases (Figure 2.)

As seen from the phylogenetic relationships of this gene, its topology supported previously obtained data on the hemagglutinin gene, indicating the division into two major H7N7 EIV clusters: the prototype-like, and a group comprising other equine viruses ascending to the early American isolates of the mid-1960s.

**Figure 3:**
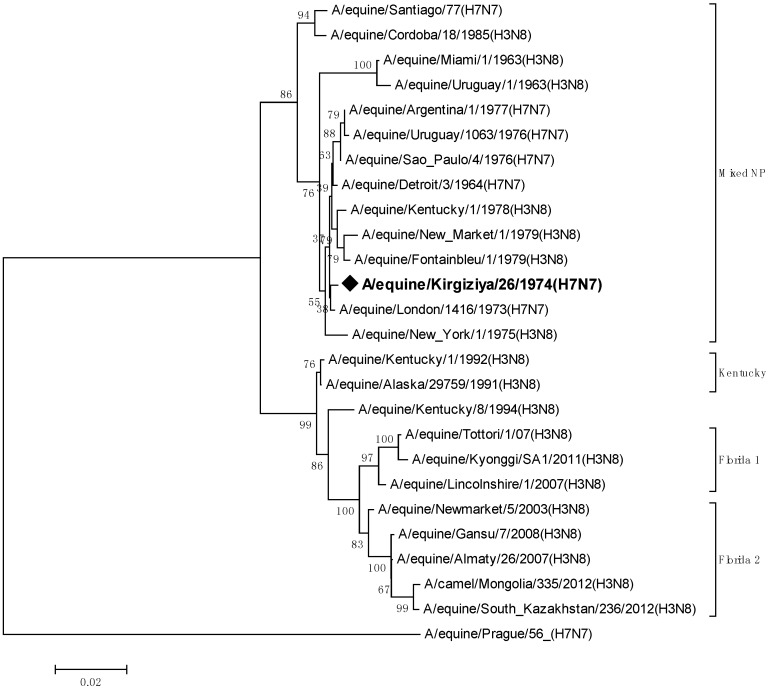
Phylogenetic tree of the nucleotide sequences of nucleoproteins of EIV belonging to H7N7 and H3N8 subtypes.

The next most important gene for genetic analyses is the nucleoprotein gene which encodes the primary protein of the nucleocapsid. Phylogenetic relationships for this gene are shown in Figure 3.

The phylogenetic tree shows that EIV NP genes have evolved into two very divergent lineages. The first one is that of Prague/56 NP, which forms a sister group to all other influenza A virus NPs. It was suggested that this NP genotype is the most primitive between all known influenza A virus NPs [14]. The second branch consists of all other known EIV strains. The strain A/equine/Kirgizia/26/1974 (H7N7) appeared to belong to the latter branch which contains both H7N7 and H3N8 serotypes. Bean et al. (1984) [15] suggested that H7N7 EIVs of the 1960s and later are reassortants that lost their initial Prague/56-like NP.

The horse population in Kyrgyzstan reaches 400,000 heads [11]. In October 2014, during the non-epidemiological season, blood and nasal swab samples from 76 unvaccinated horses were tested; EIV was not detected. Sera were tested in a hemagglutination inhibition test for antibodies to EIV subtypes (H7N7 Prague reference strain and H3N8 Wildeshausen/08). Antibodies to H7N7 were not found in the population but antibody titer to the H3N8-isolate Wildeshausen/08 in Hemagglutination Inhibition assay was 1:8 [16]. Nasal samples were inoculated into embryonated hens’ eggs for virus isolation and EIV could not be identified.

## 3. Materials and Methods

Archival virus deposited in the National Collection of Microorganisms located at the Research Institute for Biological Safety Problems in Kazakhstan was used.

Viral RNA was extracted from allantoic fluid using the QIAamp Viral RNA Mini kit (Qiagen, Hilden, Germany), according to the manufacturer’s instructions.

Reverse transcription-PCR (RT-PCR) assays were performed using segment-specific primers [17] on the basis of one-step protocols using appropriate RT-PCR Kits (One Step RT-PCR kit) according to the manufacturers’ instructions.

PCR products of the anticipated size were purified from agarose gels using the QIAquick Gel Extraction Kit (Qiagen). Purified DNA fragments were cycle-sequenced in both directions using the same primers as for RT-PCR. BigDye Terminator v3.1 cycle sequencing kit (Applied Biosystems, Darmstadt, Germany) was used and amplicons were analyzed on an automatic sequencer (ABI-3130 xl, Applied Biosystems).

Sequence of the hemagglutinin gene of the strain A/equine/Kirgizia/26/1974 (H7N7) was deposited in Genbank under accession number JX983548.

Phylogenetic trees were constructed using HA gene sequence of A/equine/Kirgizia/26/1974 (H7N7) (Figure 1) and publicly available equine H7N7 sequences (GenBank and EMBL). The trees were generated by the Neighbor-Joining method (using the Tamura-Nei model with 1000 bootstrap replicates) and confirmed with maximum likelihood to verify tree topology.

## 4. Conclusions

In conclusion, the Central Asian equine influenza virus isolate did not contain any specific genetic signatures and can be considered as an epizootic strain of 1974 that spread in Europe. It is hard to say how the European sublineage virus could reach the mountainous region of Soviet Central Asia because of the strict regime and closed borders during the communist era in Kirgizia. Unfortunately, no epidemiological data are available about this outbreak from the Soviet era. We suppose that the virus circulated in this large region, which includes Kazakhstan, Kirgizstan, China and Mongolia. The absence of antibodies to this subtype EIV in recent studies confirms its disappearance as of the 1990s when the antibodies were last found in unvaccinated horses.

## Figures and Tables

**Table 1:** Changes in amino acid residues in A/equine/Kirgizia/26/1974 (H7N7) hemagglutinin protein compared to consensuses derived from published sequences and London/73.

Isolate	Site B						Site D						Site E		Site C	
	164	168	169	198	202	208	182	183	211	220	223	253	67	96	54	277
Prototype-like consensus	S	G	I	T	R	D	K	V	I	Q	V	T	V	V	T	Q
American consensus	N	G	V	A/D	R	N/D	R	E	V	R	A	T/N	I	I	K	Q
London/73	N	G	V	A	R	N	R	E	I	Q	A	T	I	I	K	Q
**Kirgizia/74**	**D**	G	V	A	R	N	R	E	I	Q	A	T	I	I	K	Q
